# Prevalence and Antimicrobial Resistance of *Escherichia coli* O157:H7 and *Salmonella*, and the Prevalence of *Staphylococcus aureus* in Dairy Cattle and Camels under Pastoral Production System

**DOI:** 10.3390/antibiotics13010026

**Published:** 2023-12-27

**Authors:** Diriba Hunduma, Kebede Amenu, Hiwot Desta, Delia Grace, Getahun E. Agga, Oudessa Kerro Dego

**Affiliations:** 1 College of Agriculture and Environmental Sciences, Arsi University, Asella P.O. Box 193, Ethiopia; diribahunduma@gmail.com; 2 College of Veterinary Medicine and Agriculture, Addis Ababa University, Bishoftu P.O. Box 34, Ethiopia; kebede.amenu@aau.edu.et; 3 International Livestock Research Institute, Addis Ababa P.O. Box 5689, Ethiopia; h.desta@cgiar.org; 4 International Livestock Research Institute, Nairobi P.O. Box 30709, Kenya; d.randolph@cgiar.org; 5 Natural Resources Institute, University of Greenwich, Chatham Maritime, Kent ME4 4TB, UK; 6 Food Animal Environmental Systems Research Unit, Agricultural Research Service, United States Department of Agriculture, Bowling Green, KY 42101, USA; getahun.agga@usda.gov; 7 Department of Animal Science, The University of Tennessee, Knoxville, TN 37996, USA

**Keywords:** *E. coli* O157:H7, *Salmonella*, *Staphylococcus aureus*, dairy cattle, camel, milk-borne pathogen, pastoral livestock production

## Abstract

*Escherichia coli* O157:H7, *Salmonella* and *Staphylococcus aureus* are common foodborne pathogens. We determined the prevalence of *E. coli* O157:H7 and *Salmonella* in feces and milk and the prevalence of *S. aureus* in milk from dairy cattle and camels in the Borana pastoral community in the Southern Oromia Region of Ethiopia. Paired individual cow composite (pooled from all quarters in equal proportions) milk and fecal samples were collected from cows (*n* = 154) and camels (*n* = 158). Samples were cultured on bacterial isolation and identification media. *E. coli* O157:H7 and *Salmonella* isolates were further tested for susceptibility against nine antimicrobial drugs. Different risk factors associated with hygienic milking practices were recorded and analyzed for their influence on the prevalence of these bacteria in milk and feces. The prevalence of *E. coli* O157:H7 and *Salmonella* in feces were 3.9% and 8.4%, respectively, in cows, and 0.6% and 2.5%, respectively, in camels. *E. coli* O157:H7 and *Salmonella* were detected in the composite milk samples of 2.6% and 3.9% of the cows, respectively, and 0% and 1.3% of the camels, respectively. *S. aureus* was detected in composite milk samples of 33.4% of the cows and 41.7% of the camels. All *E. coli* O157:H7 (*n* = 11) and *Salmonella* (*n* = 25) isolates from both animal species and sample types were resistant to at least one antimicrobial drug. Multidrug resistance was observed in 70% (7/10) of the *E. coli* O157:H7 fecal and milk isolates from cows and 33.3% (2/6) of the *Salmonella* fecal and milk isolates from camels. The prevalence of these bacteria in feces and milk was not affected by risk factors associated with milking practices. Given the very close contact between herders and their animals and the limited availability of water for hand washing and udder cleaning, these bacteria are most likely present in all niches in the community. Improving community awareness of the need to boil milk before consumption is a realistic public health approach to reducing the risk of these bacteria.

## 1. Introduction

Milk and milk products play a significant role in human health and well-being [1,2]. However, milk-borne pathogens cause human diseases ranging from gastrointestinal disturbances such as diarrhea and vomiting to systemic and even life-threatening illnesses [3,4,5,6]. The presence of milk-borne pathogens in milk has both public health and economic importance [7,8]. The economic losses incurred by the dairy industry can be associated with reduced consumer confidence impacting the market for dairy products [9,10], product recalls, or the effects of some pathogens on animal productivity. The microbiological quality of dairy products in relation to foodborne pathogens is of great concern worldwide and is especially true in developing countries where dairy products are commonly handled under inadequate hygienic conditions and frequently consumed raw [8,11]. Milk-borne pathogens, including *Salmonella*, *E. coli* O157:H7 and *Staphylococcus aureus* can infect humans following the consumption of non-pasteurized milk and milk products [7,12]. Lack of routine milk pasteurization practices coupled with poor hygienic milk handling and processing under traditional livestock production systems is common in many developing countries [11,13,14,15,16]. In Ethiopia, a recent review of the available literature [17] indicated medians of 6% and 10% prevalences of *Salmonella* and *E. coli* O157:H7, respectively, in raw cow milk.

Most studies on *E. coli* O157:H7 in livestock species have been conducted on samples collected from different parts of beef cattle, sheep and goats at abattoirs or slaughterhouses and retail meat from different livestock species and other food samples [18,19,20,21,22,23]. The overall prevalence of *E. coli* O157:H7 in meat and other sample types was low, usually below 10%, but most of them had high antimicrobial resistance patterns, including multidrug resistance phenotypes [18,19,20,21,22,23]. Studies on *Salmonella* in Ethiopia have focused on testing the presence of *Salmonella* in different livestock species and foods of animal origin (meat and its minced products, raw eggs and raw milk), animal feces and human stool and their antimicrobial susceptibility profiles [24,25,26,27,28]. The prevalence of Salmonella is low in ruminants (cattle, sheep and goats) but high in pigs [26]. The prevalence of *Salmonella* in food of animal origin ranges from 3 to 10%, and antimicrobial drug resistance has also been observed against almost all tested antibiotics that are commonly used in both veterinary and human health sectors [24,25,27,28]. *Staphylococcus aureus* is the most common and frequently isolated bacteria responsible for mastitis, with variable prevalence in cows, and udder quarters, from different parts of Ethiopia [11,29,30,31,32,33].

Although *E. coli* O157:H7, *Salmonella* and *S. aureus* have been extensively studied in the highlands of Ethiopia [11,13,18,34,35,36,37], their statuses are not well understood in the pastoral settings where large herds of livestock are raised in extensive systems. Borana is an expansive savanna grassland in the Southern Oromia State of Ethiopia. It is characterized by an arid to semi-arid climate where the community’s livelihood mainly depends on livestock production. Milk is commonly consumed by the Borana pastoral community [38,39]. In this community, milking cows and processing milk are conducted using local traditional methods that are affected by various socio-cultural practices and beliefs [15,40]. Information on the occurrence of foodborne pathogens such as *E. coli* O157:H7 and *Salmonella* and the major milk-borne pathogen *S. aureus* in dairy animals and their milk is limited in these pastoral communities. The objective of this study was to determine the prevalence of *E. coli* O157:H7, *Salmonella* and *S. aureus* in dairy cows and camels raised under the pastoral livestock production system in Borana. Antimicrobial resistance of *E. coli* O157:H7 and *Salmonella* isolates were also determined.

## 2. Results

### 2.1. Description of the Study Animals

**Figure 1:**
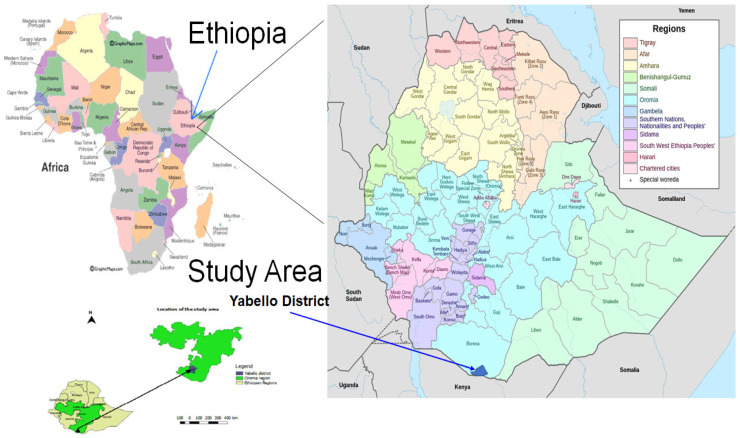
Geographical location of the study area.

Dairy Cows: Paired fecal and milk samples were collected from 154 lactating cows belonging to 96 herds in 13 villages from 4 districts in the Borana zone (Figure 1). On average, 1.6 cows were sampled per herd, with a median of 1 and a range of 1–8 cows per herd. Only 1 cow was sampled per herd in two-thirds of the herds (66.7%; *n* = 96); 2 cows were sampled in 17.7% of the herds; 3 cows were sampled in 11.5% of the herds; and in the remaining four herds (4.2%), 4, 5, 6 or 8 cows were sampled per herd. Almost all study cows (98.7%, *n* = 154) were sampled from herds that raised mixed livestock species, with two-thirds (66.9%) of the sampled cows being from herds that raised 4 livestock species (cattle, camels, goats and sheep; Table 1. The study cows were seven years old on average, with the majority (57.8%) of the cows in good condition at the time of sampling. On average, the study cows were 11.5 months in lactation, with a mean parity number of 2.6 (Table 1).

**Figure 2:**
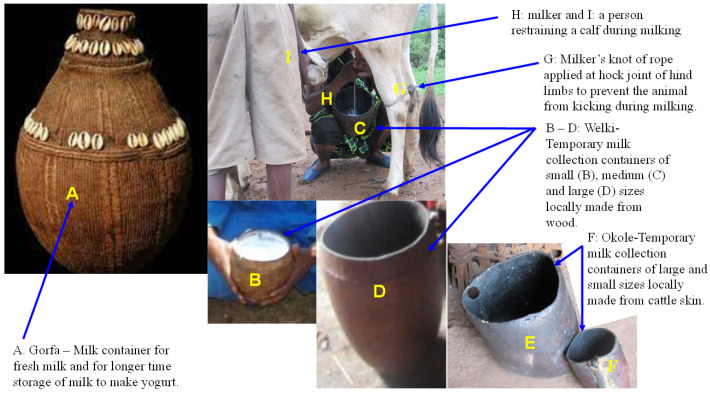
Locally made milk collection and storage containers. (**A**) Gorfa is a milk container that is handmade using traditional techniques. It is made from very tightly woven strands of vegetable or sisal fibers bunched together and wrapped at regular intervals with either one or two other fibers and decorated with cowry shells. Prior to use for milk storage, it is cleaned with water and smoked with glowing embers of local trees (Ejersa, scientific name *Olea europaea* subsp. *cuspidate*; Daanse, scientific name *Faurea speciose*; and Birreessa, scientific name *Terminalia brownie*) usually used for smoking milk containers [41]. The container is light and extremely durable and the inside has a black encrusted patina which makes it waterproof and ideal as a liquid container; (**B**–**D**) Welki is a temporary milk collection container locally made from wood and used when milking. It is available in different sizes, including small (**B**), medium (**C**) and large (**D**); (**E**,**F**) Okole is temporatry milk collection container locally made from skin of cattle and available in different sizes including large (**E**) and small (**F**); (**G**) a rope tied across both hind limbs at the hock joint using a milker’s knot to prevent the cow from kicking during milking; (**H**) a pastoralist woman kneel down on her leg and hold Welki (temporary milk collection container) tightly between her thighs and milking the cow quickly with both hands; (**I**) a person restraining the calf while the cow is milked. This picture was taken during study tim.

With regards to milking practices, over half (56.5%) of the study cows were milked into a locally made container called an “Okole”, (Figure 2E,F) which is a bucket made from the fresh skin of a giraffe or cow [41]. Another milk collection container was “Welki”, which is made locally from wood (Figure 2B–D). The rest of the cows and camels were milked into commercially available plastic buckets. Nearly all cows (91%) had relatively clean udders, and most were milked with no hand washing (80%), udder preparation (93%), or container cleaning (77%). Most cows (68%) were restrained by a rope tied to the hocks during milking (Figure 2G), and calves were allowed to suckle in more than half of the cows (58%) or restrained by a person (Figure 2I). Cows were milked primarily by women (84%; Table 1). The fecal consistency of the study cows was mostly normal or fluid and almost all cows had no teat lesions (Table 1).

Dairy Camels: paired fecal and milk samples were collected from 158 lactating camels belonging to 91 herds in 10 villages from 4 districts in the Borana zone (Table 2). On average, 1.7 camels were sampled per herd, with a median of 1 and a range of 1–4 camels per herd. Only one camel was sampled per herd in over half of the herds (53.9%; *n* = 91); two camels were sampled in 24.2% of the herds; three camels were sampled in 16.5% of the herds; and four camels were sampled per herd in the remaining five herds (5.5%). Almost all study camels (98.7%, *n* = 158) were sampled from herds that raised mixed livestock species, with the majority of the camels (86.1%) sampled being from herds that raised four livestock species (cattle, camels, goats and sheep; Table 2). The study camels were nine years old, on average, with most of the camels being in good (46%) or medium (41%) body condition at the time of sampling. On average, the study camels were 10 months in lactation, with a mean parity number of 3.1 (Table 2).

**Figure 3:**
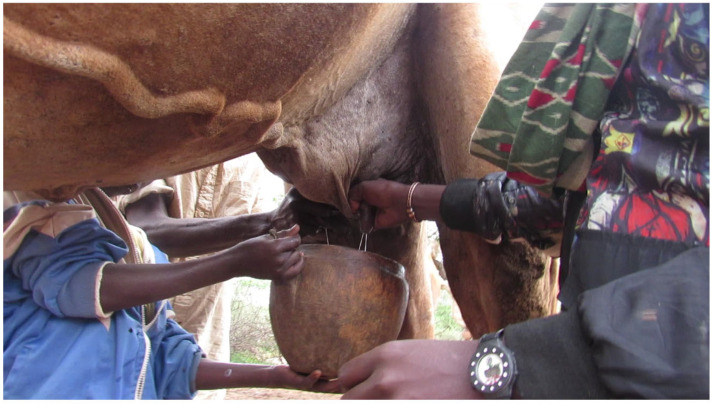
A camel milked into a Welki by three persons. A camel can be milked by two or three persons from a standing position depending on availability of person to help. If two persons are milking, one person holds the milk collection container with one hand and milks the animal with the other hand, while the second person milks the camel with both hands [42]. If three persons are milking, one person holds the milk collection container and the two persons milk the camel as shown in this figure. Milk let-down takes a shorter time and milkers milk the camel quickly and collect milk within a short time. The picture was taken during study time.

With regards to hygienic milking practices, commercially obtained plastic containers and two locally made milk collection containers, Welki (Figure 2B–D) and Okole (Figure 2E,F), were the most common milking utensils used in the community to milk the study camels. The majority of the camels (84%) had relatively clean udders, and most camels were milked with no hand washing (89%), udder preparation (96.2%) or container cleaning (87%). The overwhelming majority of the study camels (89%) were manually restrained during milking and calves were allowed to suckle in half (58%) of the camels sampled. Unlike cows, camel milking was performed by at least two people helping each other, with family members being involved in the task (Figure 3 and Table 2). The fecal consistencies of the study camels were almost equally divided between normal and hard, and almost all study camels (98%) had no teat lesions (Table 2).

### 2.2. Prevalence of E. coli O157:H7, Salmonella and Staphylococcus aureus in the Feces and Milk of Lactating Cows and Camels in the Borana Pastoralist Community

**Figure 4:**
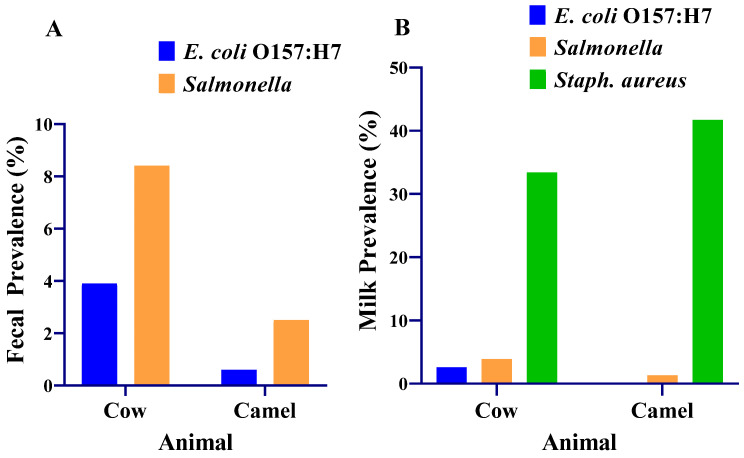
Prevalence of *E. coli* O157:H7, *Salmonella* and *Staphylococcus aureus* in the milk and feces of lactating cows and camels in the Borana pastoralist community. (**A**) Fecal prevalence of *E. coli* O157:H7 and *Salmonella*; (**B**) milk prevalence of *E. coli* O157:H7, *Salmonella* and *S. aureus*. *Staphylococcus aureus* was cultured from milk samples collected from 119 cows and 130 camels.

The prevalence of *E. coli* O157:H7 and *Salmonella* were 3.9% and 8.4%, respectively, in cow feces (Figure 4). *E. coli* O157:H7 and *Salmonella* were detected in 2.6% (4/154) and 3.9% (6/154), respectively, of composite milk samples from cows (Figure 4). All the *E. coli* O157:H7 and 77% (10/13) of the *Salmonella*-positive cows were from Yabello district. Cows that were positive for both pathogens were found in various villages in the positive districts, with most cases detected in one village (Dida Yabello). Most cases occurred in cows sampled from mixed herds raising the four livestock species: cattle, camels, goats and sheep.

The prevalence of *E. coli* O157:H7 and *Salmonella* were 0.6% and 2.5%, respectively, in the feces of camels. *E. coli* O157:H7 was not detected in any of the composite camel milk samples tested. *Salmonella* was detected in 1.3% (2/158) of composite camel milk (Figure 4).

The prevalence of *Salmonella* was significantly higher (*p* = 0.025) in the feces of cows than in the feces of camels. The prevalences of *E. coli* O157:H7 in feces were not different between cows and camels (*p* = 0.064). The prevalences of *E. coli* O157:H7 and *Salmonella* (*p* = 0.169) in composite milk did not differ significantly (*p* = 0.058) between the cows and the camels.

*S. aureus* was detected in composite milk samples of 33.4% of the cows and 41.7% of the camels. The prevalence of *S. aureus* was significantly higher (*p* = 0.026) in composite milk samples from camels (41.7%) than in composite milk samples from cows (33.4%).

#### 2.2.1. Association between Risk Factors and *E. coli* O157:H7, *Salmonella* and *S. aureus* Prevalence in Cow Feces and Milk

Prevalence of *E. coli* O157:H7 in fecal samples: Study district was significantly associated with the prevalence of both *E. coli* O157:H7 (*p* = 0.02) and *Salmonella* (*p* = 0.019) in the feces of cows (Table 3).

As shown in Table 3, the prevalence of *E. coli* O157:H7 in feces was not significantly affected by age (*p* = 0.575), body condition score (*p* = 0.641), stage of lactation at sampling (*p* = 0.575), or parity number (*p* = 0.407) of the cow. Similarly, *E. coli* O157:H7 in the feces was not significantly affected by the type of container used for milking (*p* = 0.414), the person milking the cow (*p* = 1.00), whether or not hands were washed before milking (*p* = 0.575), whether or not the milk container was washed before milking (*p* = 0.338), by the type of restraining method used during milking (*p* = 1.00), or whether or not calves were allowed to suckle during milking (*p* = 0.693). *E. coli* O157:H7 was not significantly affected by the fecal consistency (*p* = 0.398) or the presence of teat lesions (*p* = 0.182).

The 6 positive fecal samples were obtained from cows that were 5 (1 sample), 6 (3), 8 (1) and 10 (1) years old. Five of the six positive samples were obtained from cows in good body condition. Five positive fecal samples were obtained from cows in late lactation (12 months in lactation) and a single positive sample was obtained from an early lactating cow (2 months in lactation). Five of the six positive fecal samples were obtained from cows between 1 and 3 parities, while four of the positive fecal samples were obtained from cows milked into plastic containers and the remaining two samples were obtained from cows milked into Okole. We noted that all positive fecal samples were obtained from cows that were milked without hand washing. Four of the positive fecal samples were obtained from cows restrained by ropes tied to the hocks during milking. Five of the six positive fecal samples were obtained from cows with normal fecal consistency. Similarly, five of the six positive fecal samples were obtained from cows with no teat lesions.

Prevalence of *Salmonella* in feces: The prevalence of *Salmonella* in cow feces (Table 3) was not significantly affected by the age (*p* = 0.665), body condition score (*p* = 0.536), stage of lactation at sampling (*p* = 0.545), or parity number (*p* = 0.645) of the cow. Similarly, the prevalence of *Salmonella* in the feces was not significantly affected by the type of container used for milking (*p* = 0.939), the person milking the cow (*p* = 0.452), whether or not hands were washed before milking (*p* = 0.468), whether or not the milk container was washed before milking (*p* = 0.3), or by the type of restraining method used during milking (*p* = 1.00). Calf suckling before milking significantly reduced the prevalence of *Salmonella* in cow feces (*p* = 0.042); the prevalence of *Salmonella* in the feces was 14.1% (*n* = 64) in calf-suckled cows versus 4.4% (*n* = 90) in cows milked without calf suckling. The prevalence of *Salmonella* in the feces was not significantly affected by fecal consistency (*p* = 0.077) or the presence of teat lesions (*p* = 0.100).

*Salmonella* was detected in cows aged between 5 and 9 years old, with most (46.2%, *n* = 13) detected in 8-year-old cows. Positive samples were obtained from cows in good or medium body condition. Most positive samples (10/13) were obtained from cows in late lactation (12–24 months in lactation), with the remaining 3 positive samples coming from early lactating cows (1–6 months in lactation). The positive fecal samples were obtained from cows with parities between 1 and 4. Most positive fecal samples were obtained from cows milked into Okole (7 positives) or plastic containers (5 positives), while the one remaining positive sample was from a cow milked into a jerrycan. All positive cows were milked by women. We noted that 12 of the 13 positive fecal samples were obtained from cows that were milked without washing hands and without cleaning containers. Nine of the positive fecal samples were obtained from cows restrained using ropes tied to the hocks during milking, while the remaining four were from cows manually restrained. Nine of the thirteen positive fecal samples were obtained from cows with normal fecal consistency, three were from cows with fluid fecal consistency and the remaining one positive sample was from a cow with a hard fecal consistency. All *Salmonella*-positive fecal samples were obtained from cows with no teat lesions.

*E. coli* O157:H7 in composite milk: Overall, four composite milk samples from the dairy cows were positive for *E. coli* O157:H7. *E. coli* O157:H7 positivity was not significantly (*p* > 0.05) associated with any of the risk factors included in the analysis (Table 4). However, there were some notable observations within the categories of risk factors.

District was not significant (*p* = 0.089), but all four positive *E. coli* O157:H7 composite milk samples came from Yabello only. Village was not significant (*p* = 0.154), with *E. coli* O157:H7 occurring in only two of the villages: Dharito (three of the four positive composite milk samples) and Colqasa (one positive sample). All four *E. coli* O157:H7-positive composite milk samples were obtained from mixed herds that raised all four livestock species (cattle, camels, goats, sheep), although the factor was not significant (*p* = 0.78). The age of the cow was not significantly associated with *E. coli* O157:H7 positivity (*p* = 0.623), although three of the four positive composite milk samples were from 8-year-old cows. All four positive composite milk samples were obtained from cows in good BCS and lactating for 12 months, with no significant effects of BCS (*p* = 0.383) or stage of lactation (*p* = 0.904) on the detection of *E. coli* O157:H7 in composite cow milk. The effect of parity was not significant (*p* = 0.415); one positive milk sample each was obtained from cows in their 1st and 3rd parity, while the remaining two positive samples were from cows in their 4th parity. Two each of the four positive composite milk samples were obtained from cows milked into Okole or plastic containers, with no significant effect of milking utensils (*p* = 0.807). The person milking the cow did not have any significant effect (*p* = 1.00), with all composite milk samples being obtained from cows milked by women. Hand washing (*p* = 0.584) and container cleaning (*p* = 0.575) before milking were not significant, although all four *E. coli* O157:H7-positive composite milk samples were obtained from cows milked without hand washing or container cleaning. The type of restraint was not significant (*p* = 1.00); three positive milk samples were obtained from cows milked following restraint with a rope. Three of the positive composite milk samples were obtained from cows that were milked after calf suckling (*p* = 0.642), from cows with fluid fecal consistency (*p* = 0.359) and from cows with no teat lesions (*p* = 0.125), although these factors were not significant. All four positive composite milk samples were obtained from cows that were milked without udder preparation (*p* = 1.00) and from cows that had relatively clean udders (*p* = 1.00).

*Salmonella* in composite milk: Overall, six *Salmonella*-positive composite milk samples were obtained from the cows sampled. Except for village (*p* < 0.001) and parity number (*p* = 0.002), all other factors were not significantly associated (*p* > 0.05) with the detection of *Salmonella* from the composite milk samples of dairy cows (Table 4). Although district was not significant (*p* = 0.382), *Salmonella* was detected in composite milk samples from cows in two of the three districts (Dubuluk and Yabello), with three positive samples each.

*Staphylococcus aureus* in composite milk: Most of the risk factors were not significantly associated (*p* > 0.05) with the prevalence of *S. aureus* in cows (Table 5). In the composite milk samples collected from cows, only village (*p* = 0.051) and fecal consistency (*p* = 0.002) were significantly associated with *S. aureus* prevalence.

#### 2.2.2. Association between Risk Factors and *E. coli* O157:H7, *Salmonella* and *S. aureus* Prevalence in Camel Feces and Milk

Prevalence of *E. coli* O157:H7 in fecal samples: The effects of the various studied risk factors on the prevalence of *E. coli* O157:H7 and *Salmonella* in camel feces are presented in Table 6. The single fecal sample that was positive for *E. coli* O157:H7 was obtained from a 10-year-old camel (with no age effect; *p* = 1.00) with a medium body condition score (*p* = 0.538), in her 7th month of lactation (*p* = 0.032) and her third parity (*p* = 1.00), who was milked into an Okole (*p* = 0.608) by a man and a woman (*p* = 0.31). The cow was milked after handwashing (*p* = 0.108) and cleaning the milking utensil (*p* = 0.133), and she was manually restrained (*p* = 1.00). Her calf was allowed to suckle (*p* = 1.00), her feces had a hard consistency (*p* = 0.513) and she had no teat lesions (*p* = 1.00).

Prevalence of *Salmonella* in fecal samples: All the risk factors analyzed were not significantly associated (*p* > 0.05) with the prevalence of *Salmonella* in fecal samples of lactating camels (Table 6). However, it is worth mentioning the following observed trends within the categories of each risk factor. Two of the four positive fecal samples were from 6- and 8-year-old camels, with no significant age effect (*p* = 0.4). Three positive fecal samples were obtained from camels in good BCS, with the remaining one positive sample coming from a camel with poor BCS; however, BCS was not significantly associated with the prevalence of *Salmonella* in feces (*p* = 0.234). The stage of lactation was not significant (*p* = 0.073); one *Salmonella*-positive feces sample was obtained from an early lactating camel (2 months in lactation), while three *Salmonella*-positive feces samples were obtained from late lactating camels (4–5 and 7 months in lactation). The parity number was not significant (*p* = 0.605); two *Salmonella*-positive camels were in their first parity, while the remaining two camels were in their 2nd and 4th parities. The milking container used was not significantly associated with the prevalence of *Salmonella* in feces (*p* = 0.714); two *Salmonella*-positive camels were milked into an Okole or a plastic container, while the other two camels were milked into Welki. The prevalence of *Salmonella* in fecal samples was not associated with the person(s) milking the camel (*p* = 0.131); all *Salmonella*-positive camels were milked by different people. Handwashing and container cleaning before milking were not significantly associated with the prevalence of *Salmonella* in fecal samples (*p* = 1.00), although all *Salmonella*-positive camels were milked without hand washing or container cleaning. Three of the four positive samples were obtained from manually restrained camels, although the restraint type was not significantly associated with the prevalence of *Salmonella* in fecal samples (*p* = 0.369). Three of the four camels were milked without calf suckling, although calf suckling was not significantly associated with the prevalence of *Salmonella* in fecal samples (*p* = 0.620). Fecal consistency was not significantly associated with the prevalence of *Salmonella* in feces (*p* = 1.00); two *Salmonella*-positive samples were obtained from camels with hard and normal fecal consistency each. Teat lesion was not significantly associated with the prevalence of *Salmonella* in feces (*p* = 1.00), although all four *Salmonella*-positive camels had no teat lesions. Although udder preparation did not have any significant effect on the prevalence of *Salmonella* in feces (*p* = 1.00), all *Salmonella*-positive camels did not undergo udder preparation prior to milking. Three of the *Salmonella*-positive camels had relatively clean udders, although this was not significant (*p* = 0.502).

*Salmonella* and *S. aureus* in composite milk: *Salmonella* was detected in two composite milk samples from the camels, but its detection was not significantly associated with any of the risk factors analyzed (*p* > 0.05; Table 6). Most of the risk factors were not significantly associated with the prevalence of *S. aureus* in camels (*p* > 0.05; Table 5), and district was the only risk factor that was significantly associated with *S. aureus* prevalence in composite milk samples from camels (*p* = 0.036).

### 2.3. Antimicrobial Resistance of E. coli O157:H7 and Salmonella

Antimicrobial susceptibility testing was performed for 11 *E. coli* O157:H7 isolates (10 fecal and milk samples from cows and one fecal sample from a camel) and 25 *Salmonella* isolates (19 fecal and milk samples from cows and 6 fecal and milk samples from camels). Antimicrobial susceptibility test results for *E. coli* O157:H7 and *Salmonella* isolates from milk and fecal samples for nine antimicrobial agents are shown in Table 7. Inhibition zone diameters for the antimicrobials on the test panel are provided in Table 7 and Supplementary Table S1. The isolates showed varying degrees of susceptibility to the antimicrobial agents tested. All *E. coli* O157:H7 isolates were susceptible to nalidixic acid, gentamicin, ciprofloxacin and chloramphenicol. Antimicrobial resistance of *E. coli* O157:H7 isolates was observed against ampicillin (100% of the isolates), streptomycin (73%), tetracycline (64%) and trimethoprim (18.2%). All *Salmonella* isolates were susceptible to nalidixic acid, gentamicin, ciprofloxacin and trimethoprim. On the other hand, *Salmonella* isolates were resistant to ampicillin (100% of the isolates), streptomycin (28%), kanamycin (4%) and tetracycline (12%) (Table 8).

All *E. coli* O157:H7 isolates from fecal and milk samples from cows were resistant to at least one antimicrobial agent. Multidrug resistance (MDR), defined as resistance to ≥3 antimicrobial classes [43], was observed in 70% (7/10) of the *E. coli* O157:H7 isolates from fecal and milk samples of cows. The single *E. coli* O157:H7 isolate from camel feces was resistant only to ampicillin. *Salmonella* isolates from fecal and milk samples from cows were resistant to ampicillin alone (79%) or co-resistant to one or two drugs in two other antimicrobial classes (21%). While MDR was not observed in the cow isolates, 33.3% (2/6) of the *Salmonella* isolates from camel milk and feces showed MDR (Table 9).

## 3. Discussion

The present study was conducted as part of a milk hygiene improvement research project in the Borana pastoral communities [13,44] to determine the prevalence of *E. coli* O157:H7 and *Salmonella* (in both feces and milk) and *S. aureus* (in composite milk only) in lactating cows and camels. Studies focusing on the prevalence of these pathogens in lactating dairy animals are scarce [45,46] and the available ones were mainly conducted in the central highlands of Ethiopia and focused primarily on animals destined for slaughter at abattoirs [18,47,48,49,50].

The 3.9% prevalence of *E. coli* O157:H7 in fecal samples from cows is comparable to the prevalence observed in cattle feces from abattoirs in Ethiopia (4.7%) [50] and Qatar (5%) [51]. On the other hand, a lower prevalence (1.9%) of *E. coli* O157:H7 was reported in central Ethiopia [18]. Compared to the present study, a higher prevalence (10.7%) of *E. coli* O157:H7 in cattle feces was reported in Riyadh, Saudi Arabia [52]. The same study [52] also reported a 2.4% prevalence of *E. coli* O157:H7 in camel feces, which is higher than the 0.6% prevalence observed in our study. Similar to the present study, low prevalences (1% [51] and 0.6% [53] of *E. coli* O157:H7 in camels were also reported elsewhere. The absence of *E. coli* O157:H7 in camel milk in the present study is contrary to a previous study from Qatar, which reported a high occurrence of *E. coli* O157:H7 (34%, *n* = 50) in camel fecal samples [51].

In the present study, the 8.4% prevalence of *Salmonella* in fecal samples from cows is higher than the 2.3% prevalence [46] but nearly similar to the 7.7% prevalence reported in dairy farms in Addis Ababa [45]. Farm-level contamination of cow milk with *Salmonella* in the present study (3.9%) is similar to the 3.1% prevalence in a previous report from central Ethiopia [45]. However, making these valid comparisons can be difficult given that most of the previous studies mostly involved cattle bound for slaughter after transportation from their initial production sites. Stress due to transportation can increase pathogen shedding. In the present study, on-farm samples were collected from animals raised under natural conditions in an extensive livestock production system.

The results of the present study showed that considerable proportions of the raw milk sampled from cows and camels at the farm level (primary production) were positive for *Salmonella* and *E. coli* O157:H7. Under such circumstances, the pathogens can present public health risks given that raw milk consumption is common in the area [15] and that attitudinal changes from this practice were not sustained after public education [13]. Further, the risk is potentiated by the ability of *E. coli* O157:H7 to survive harsh conditions, such as the low pH of dairy products [54]. We noted that under such subsistence farming, milk production is primarily for household consumption, with little sold to meet the financial needs of the family. There are no refrigeration or pasteurization facilities in this area and, as such, milk is consumed raw, posing a significant risk to consumers, especially children.

Risk factors such as pre-milking teat washing, milkers’ hand washing, presence of trauma/injury on teats, pre-cleaning of milk collection containers, milkers (male or female), animal body condition and fecal consistency were collected and their effects on the prevalence of these bacteria were analyzed in fecal and milk samples (Table 1 and Table 2). None of these risk factors significantly influenced the prevalence of these pathogens in fecal and milk samples. Given the fact that none of these hygienic milking practices were used in these areas before and that producers lack enough water for cleaning and have limited experience with hygienic milking procedures, it is not surprising to find no effects of these risk factors. In the absence of basic access to clean water and toilets in pastoral communities, and widely practiced raw milk consumption and close human–animal contact, the prevalence of these pathogens in feces, milk and other niches in these pastoral communities may not vary. However, there are no widespread milk-borne illnesses due to these pathogens. It is not clear whether this is due to adaptation to these pathogens due to frequent exposure early on or whether it is due to other mechanisms.

The current study on the antimicrobial susceptibility of *E. coli* O157:H7 and *Salmonella* revealed varying degrees of susceptibility to the antimicrobial agents tested. The degrees of susceptibility of *E. coli* O157:H7 and *Salmonella* isolates to specific antimicrobials varied from 0% to 100%. All isolates, from both cows and camels, were 100% susceptible to nalidixic acid, gentamicin and ciprofloxacin, which is in agreement with previous studies in Ethiopia [50]. The finding that all isolates were 100% resistant to ampicillin is in line with a previous study [50] and may indicate the widespread use of this antimicrobial in pastoral communities, mainly for the treatment of mastitis in dairy animals [11]. A similar study [45] in central Ethiopia also indicated resistance of *Salmonella* isolates to commonly used antimicrobials, including ampicillin (100%), streptomycin (66.7%), nitrofurantoin (58.3%), kanamycin and tetracycline (33.3%).

In conclusion, *E. coli* O157:H7 and *Salmonella* were detected in the milk and feces of a considerable number of lactating cows. Similarly, *S. aureus* was detected in milk of lactating cows and camels. The presence of these pathogens in cow milk indicates that they were shedding through milk from infected gland or contaminated either by infected cows or unhygienic conditions during milking and handling at the level of primary production. This is particularly important in causing potential health effects in people who commonly consume raw milk and milk products. Moreover, the occurrence of multidrug-resistant *E. coli* O157:H7 and *Salmonella* in the feces and milk of lactating cows can pose a significant public health risk. Therefore, relevant intervention programs and the creation of awareness on best practices for milk handling as well as control and surveillance programs for antimicrobial usage in animals can be implemented to minimize the contamination of milk and milk products with antimicrobial-resistant pathogens.

As a limitation, we did not conduct whole genome sequencing and comparative analyses of the bacterial isolates obtained from milk and feces to determine whether the isolates were genetically identical but contaminating different samples or whether they were genetically different. Additionally, further detailed investigations are required to understand short-term and long-term health-related problems or impact caused by frequent exposure of public especially children at early age in life to these foodborne pathogens in this pastoral community.

## 4. Materials and Methods

### 4.1. Study Area

The study was conducted in selected villages in four districts (Yabello, Surupha, Dubuluk and Elweya) of the Borana zone, Oromia (Figure 1). These villages were selected based on their high milk production potential and ease of accessibility via cars. Borana zone is located in the lowlands of the Southern part of Oromia, Ethiopia. Yabello is the capital city of Borana zone and is about 570 km from Addis Ababa (Figure 1). The Borana pastoral area has a semi-arid to arid climate with dry and rainy seasons and bimodal rainfall distribution consisting of a long rainy season from March to May and a short rainy season from September to November. Despite usually expecting two rainy seasons, rainfall is increasingly becoming erratic and highly variable, resulting in frequent droughts and variability in livestock and livestock products off-take. The Borana community comprises both pastoral (those who only raise livestock) and agropastoral (those who grow some crops and also raise livestock) communities. Livestock production is a major source of livelihood for the community. Borana pastoralists historically raise only cattle; however, due to recent increasing erratic rainfall and drought problems, they have diversified their herds by additionally raising more drought-resilient livestock, including camels, goats and sheep [39]. The study area is typical of other pastoral settings where communities heavily depend on animal production usually raised comingled together or mixed species (cattle, sheep, goats and camels) [13,15]. People, domestic animals and wild animals share spaces and drinking water and live in close contact, which may favor the cross-species transmission of many infectious diseases, including the foodborne pathogens targeted in this study. Moreover, this study area is close to the border with Northern Kenya and Somalia and there is frequent cross-border contact between herders through grazing lands, livestock trade business as well as animal and human drugs smuggled across borders. Additional description of the study area is available elsewhere [13,15].

### 4.2. Study Design and Sample Size Calculation

A cross-sectional study was conducted in April 2018 to determine the prevalence of *E. coli* O157:H7 and *Salmonella* in the feces and milk and *S. aureus* in milk of dairy cows and camels. The study population comprised healthy-looking lactating cows and camels managed under a traditional/extensive husbandry system in the study area. Convenience sampling was used to select an individual animal from each herd. Paired fecal and milk samples were collected from each animal to determine the apparent prevalence of the target pathogens. The number of animals required to estimate prevalence was calculated using the following formula, which has been described elsewhere [55]:

$$N=\frac{{\left(Z\alpha /2\right)}^{2}\times p(1-p)}{d^{2}}$$

where N is the required sample size, *d* is absolute precision (*d* = 0.05), and *p* is expected prevalence. The prevalence (*p*) used in the sample size calculation was a pooled prevalence of 7.47% obtained from a meta-analysis of *Salmonella* in ruminants in Ethiopia [26].

Accordingly, 106 camels and 106 cows were needed, assuming equal sample sizes for *E. coli* O157:H7, *Salmonella* and *S. aureus*. However, to account for herd-level clustering of bacterial infection and contamination, the target sample size was adjusted for an intra-cluster correlation coefficient (ρ) of 0.2 [56], and about 1–8 animals were sampled per herd. The study’s design effect (*deff*), calculated as *deff* = 1 + (m − 1)ρ, where m = 3 is the cluster size and ρ = 0.2 is the correlation coefficient, was 1.4. Therefore, the sample size obtained using a simple random sampling formula was adjusted by multiplying it by the *deff*, resulting in 149 cows and 149 camels. In the end, to account for any potential sample losses, paired fecal and milk samples were collected from 154 lactating cows and 158 camels.

### 4.3. Milk and Fecal Sample Collection and Transportation

Fecal samples (~15 g) were collected rectally from individual animals using a gloved hand while the animals were restrained. A 30 mL sample of composite milk (pooled milk from all quarters) was collected from each animal. Prior to sample collection into sterile tubes, milk was collected from each animal either into commercially obtained plastic containers or locally made milk collection containers (Okole, made from cattle hide, or Welki, made from wood) (Figure 2). Samples were collected either early in the morning (around 5 a.m.) before the animals were released to pasture or after 5 p.m. in the evening when animals returned to their housing. Composite milk and fecal samples were collected in sterile bottles labeled with unique animal identifier numbers consisting of animal species, herd and sampling date. “Okole” is a bucket made from the fresh skin of a giraffe or cow. Samples were kept at +4 °C and transported to the microbiology laboratory at the International Livestock Research Institute (ILRI) in Addis Ababa, Ethiopia. Samples were stored at −20 °C until processed for microbiological analysis. During field sampling, data on potential risk factors associated with milking and hygienic practices such as the containers used for milk collection, the presence of trauma on teats or udders, whether milker(s) washed their hands, the udders and milk collection containers before milking, the animal restraining methods used during milking, the sex (male or female) and total number of milkers, fecal consistency on the day of milk collection and overall body condition of each animal were also collected.

### 4.4. Bacterial Isolation and Identification

#### 4.4.1. *Salmonella* spp. and *E. coli* O157:H7

Isolation and identification of the bacteria was done using standard techniques recommended by the International Organizations for Standardization [57] with some modifications [58]. Samples were pre-enriched by mixing 10 g of feces or 10 mL of milk with 90 mL of buffered peptone water (BPW; Oxoid, Basingstoke, UK) in Whirl-Pak filter bags (Thomas Scientific, Houston, TX, USA). The mixture was homogenized in a laboratory blender (Oxoid). Pre-enrichments were incubated at 25 °C for 2 h, then at 42 °C for 6 h and held at +4 °C until they were processed the next day for isolation of *E. coli* O157:H7 and *Salmonella*.

Pre-enrichment broth (1 mL) was added to 20 µL of anti-*E. coli* O157:H7 immunomagnetic separation (IMS) beads for *E. coli* O157:H7 isolation (Dynabeads anti-*E. coli* O157:H7; Applied Biosystems, Foster, CA, USA) or 20 µL of *Salmonella*-specific IMS beads for *Salmonella* isolation (Dynal, Lake Success, NY, USA) as previously described [59,60]. Briefly, *E. coli* O157:H7- and *Salmonella*-specific IMS beads were re-suspended by gently vortexing the mixture to ensure that the pellet was completely suspended. Twenty microliters (20 µL) of re-suspended paramagnetic beads was transferred to Eppendorf tubes (Oxoid) and 1 mL of the enriched culture was added into the Eppendorf tubes. Each tube was vortexed for 10–30 min at room temperature. Tubes were then transferred to a manual magnetic particle concentrator (MPC-S; Oxoid) with a magnetic strip in place, inverted several times and left to separate for 3 min. The supernatant was aspirated and discarded. The magnetic strip was removed and 1 mL of phosphate buffered saline with Tween 20 (PBS-T; Sigma chemical Co., Saint Louis, MO, USA) was added to each tube. The beads were re-suspended by inverting MPC several times with the tubes still in place. The magnetic strip was replaced and the above steps were repeated three times. To prevent cross-contamination, separate sterile micropipette tips were used for each sample.

The final bead–bacteria complexes (50 µL) were plated on CHROMagar O157 plates (CHROMAgar-O157:H7; DRG International, Mountainside, NJ, USA) supplemented with novobiocin (5 mg/L) and potassium tellurite (2.5 mg/L; Sigma chemical Co) and incubated at 37 °C overnight for the isolation of *E. coli* O157:H7. Following incubation, presumptive *E. coli* O157:H7 colonies with a mauve-pink color on the CHROMAgar plates were picked and inoculated on nutrient agar slants and incubated at 37 °C for 18 h. Slants were stored at +4 °C until biochemical tests were performed.

For the isolation of *Salmonella*, bacteria–bead complexes were eluted into 3 mL of Rappaport Vassiliadis soya peptone broth (RVS; Oxoid) and incubated at 42 °C for 18 h. After incubation, a loopful of RVS broth enrichment culture was plated onto xylose lysine deoxycholate (XLD) agar (Oxoid) supplemented with 4.6 mL/L tergitol), 15 mg/L novobiocin and 5 mg/L cefsulodin (XLDtnc; Sigma chemical Co.) and incubated at 37 °C for 18 h. A suspected *Salmonella* colony based on characteristic appearance on the XLD plate was inoculated on nutrient agar slants and incubated at 37 °C for 18 h. Slants were stored at +4 °C until biochemical tests were performed.

For biochemical tests, colonies were re-streaked on nutrient agar (Oxoid) plates and incubated at 37 °C for 24 h. *E. coli* O157:H7 and *Salmonella* isolates were biochemically tested using triple sugar iron agar (TSI; Oxoid), L-lysine decarboxylation test, indole production, citrate utilization test and methyl red (MR) and Voges Proskauer (VP) tests. Pure colonies from nutrient agar plates were picked and inoculated in biochemical test tubes containing TSI agar, lysine decarboxylase broth, Simon’s citrate agar and tryptone broth, and incubated at 37 °C for 24 h (more than 24 h incubation was needed for the citrate utilization test) [57]. Colonies producing an alkaline slant with acid (yellow color) butt on TSI with hydrogen sulfide and gas production, formation of purple/pink color of L-lysine decarboxylation broth, color change in Simon’s citrate agar from green to blue, positive for MR test, negative for VP test and negative for tryptophan utilization (yellow–brown ring) indicating the absence of indole production were considered *Salmonella* positive. Isolates positive for indole, negative for citrate utilization, negative for VP and positive for L-lysine decarboxylation were presumptively considered to be *E. coli* O157:H7. *E. coli* O157:H7 isolates were further confirmed using a latex agglutination test with the O157:H7 antigen (Remel, Lenexa, KS, USA), following the manufacturer’s instruction.

#### 4.4.2. *Staphylococcus aureus*

*S. aureus* was isolated from milk samples according to ISO 6888-1 [61] using Baird-Parker agar (Oxoid). The methodology was modified to follow only qualitative detection of the pathogen. Egg emulsion was prepared locally from fresh chicken eggs with intact shells purchased from a local market in Addis Ababa. The eggs were cleaned with a brush using a liquid detergent and rinsed under running water. The eggshells were disinfected by immersing them in 70% ethanol for 30 s and then air drying. Each egg was broken under aseptic conditions (in the biosafety hood) and the yolk separated from the white via repeated transfer of the yolk from one half of the shell to the other. The yolk was placed in a sterile flask and sterile water was added at four times the volume and mixed thoroughly. The mixture was heated in a water bath set at 47 °C for 2 h and then kept at +3 °C ± 2 °C for 18–24 h to allow for precipitate formation. The supernatant liquid was aseptically collected into a fresh sterile flask for use. The emulsion was stored at +3 °C ± 2 °C for a maximum of 72 h.

Sixty-three grams of agar (Oxoid) was added to one liter of distilled water and boiled to dissolve the medium; this was then autoclaved at 121 °C for 15 min. After the agar was cooled to 50 °C, 50 mL of egg yolk emulsion and 3.5% potassium tellurite solution (Oxoid) were aseptically added proportionally. The mixture of molten agar was added to sterile petri dishes and allowed to solidify and then kept under sterile conditions until use. Immediately before use, the surface of the plate was dried and 0.1 mL aliquot of milk was spread using a sterile wire loop. The plate was incubated at 37 °C for 24 h and checked for typical *S. aureus* colonies. Negative plates were incubated for up to 48 h. Each plate was examined for typical *S. aureus* colonies, which appear as black colonies surrounded by a clear zone. Typical colonies were selected and sub-cultured on tryptone soya yeast extract agar (TSYEA) and incubated at 37 °C for 24–48 h for purity. The presumptive pure colony was inoculated on TSAYE agar and incubated at 37 °C overnight. Finally, the pure colony was inoculated in TSAYE broth, incubated overnight and then stored at −80 °C in sterile 85% glycerol at a proportion of 500 µL culture and 500 µL glycerol at the Forage and Feed Development Lab at ILRI.

### 4.5. Antimicrobial Susceptibility Testing for E. coli O157:H7 and Salmonella Isolates

Antimicrobial susceptibility testing was performed according to the Clinical and Laboratory Standards Institute [62] using the Kirby–Bauer disk diffusion method. The antimicrobial disks were obtained from HIMEDIA (Mumbai, India); their list, disk concentration and CLSI interpretation breakpoints are shown in Table 1. These antimicrobials were selected based on their availability in the study area and the possibility of use by herders in the study areas. Pure colony grown on nutrient agar was transferred to a 5 mL tryptone soya broth (TSB; Oxoid) and incubated at 37 °C for 18 h until growth reached 0.5 McFarland turbidity standards (Oxoid). A sterile cotton swab was dipped into the suspension and swabbed uniformly in three directions over the surface of Mueller–Hinton agar plates (Oxoid) and kept at room temperature for 30 min to allow drying. Antibiotic disks were placed on the inoculated plates using sterile forceps by gently pressing onto the agar to ensure firm contact on the surface and incubated at 37 °C for 24 h. After incubation, the diameters of the zone of inhibition were measured using a caliper and compared with CLSI [62] zone size interpretative guidelines for the family of *Enterobacteriaceae* as sensitive, intermediate or resistant (Table 1).

### 4.6. Data Analysis

Data were recorded in Microsoft Excel (Redmond, WA, USA) and cleaned for any entry errors. Data were analyzed in STATA, version 16 (StataCorp LLC, College Station, TX, USA). Descriptive statistics such as frequencies were used to estimate the prevalence of the pathogens in both composite milk and feces samples. Univariate analysis of the association between pathogen presence and potential risk factors was conducted using Fisher’s exact or chi-squared tests. A *p*-value < 0.05 (hereafter simply presented as *P*) was interpreted as a statistically significant association.

## Figures and Tables

**Table 1:** Description of the study cow population sampled for paired fecal and milk samples in the Borana pastoral community.

Parameter	Categories	No. Sampled (*n* = 154)
District	Village	
Dubuluk (*n* = 50)	Buya	3
	Igo	10
	Jigesa	2
	Lafto	22
	Malicha Huka	10
	Surupha	3
Elweya (*n* = 38)	Areri	19
	Elweya	7
	Golba	12
Yabello (*n* = 66)	Colqasa	3
	Dharito	31
	Dida Yabello	31
	Jijidu	1
Animal species raised	Cattle	2 (1.3)
	Cattle, camel	3 (2.0)
	Cattle, goat	3 (2.0)
	Cattle, sheep	2 (1.3)
	Cattle, camel, goat	13 (8.4)
	Cattle, goat, sheep	28 (18.2)
	Cattle, camel, goat, sheep	103 (66.9)
Age in years, mean (range)		7 (range: 4–13)
Body condition score	Good	89 (57.8%)
	Medium	53 (34.4%)
	Poor	12 (7.8%)
Stage of lactation in months, mean (range)		11.5 (1–24)
Parity number, mean (median; range)		2.6 (2; 1–7)
Milking utensils	Dhamela/Gorfa	1 (0.7)
	Jerrycan	14 (9.1)
	Metal cup	3 (2.0)
	Okole	87 (56.5)
	Plastic container	41 (26.6)
	Welki	3 (2.0)
	Wooden bucket	5 (3.3)
Hand washing	No	123 (79.9)
	Yes	31 (20.1)
Milking utensil cleaning	No	119 (77.3)
	Yes	35 (22.7)
Udder preparation	No	143 (92.9)
	Yes	11 (7.1)
Udder hygiene	Relatively clean	140 (90.9)
	Visibly dirty	14 (9.1)
Restraining method	Rope tying the hock	105 (68.2)
	Manually handled	49 (31.8)
Calf suckles	Yes	90 (58.4)
	No	64 (41.6)
Milker	Boy	3 (2.0)
	Girl	20 (13.0)
	Man	1 (0.7)
	Woman	130 (84.4)
Fecal consistency	Fluid	49 (31.8)
	Hard	3 (2.0)
	Normal	75 (48.7)
	Soft	27 (17.5)
Teat lesion	Yes	5 (3.3)
	No	149 (96.8)

**Table 2:** Description of the study camel population sampled for paired fecal and composite milk samples in the Borana pastoral community.

Parameter	Categories	No. Sampled (*n* = 158)
District	Village	
Dubuluk	Jigesa	30
	Lafto	12
Elweya	Areri	18
	Elweya	17
	Golba	6
Surupha	Buya	9
Yabello	Colqasa	13
	Dharito	32
	Dida Yabello	12
	Jijidu	9
Animal species raised	Camel	2 (1.3)
	Camel, goat	4 (2.5)
	Cattle, camel	9 (5.7)
	Camel, goat, sheep	1 (0.6)
	Cattle, camel, goat	6 (3.8)
	Cattle, camel, goat, sheep	136 (86.1)
Age in years, mean (range)		8.8 (range: 5–15)
Body condition score	Good	73 (46.2)
	Medium	65 (41.1)
	Poor	20 (12.7)
Stage of lactation in months, mean (range)		10.4 (1–24)
Parity number, mean (median; range)		3.1 (3; 1–10)
Milking utensils	Dhamela/Gorfa	4 (2.5)
	Jerrycan	2 (1.3)
	Metal cup	8 (5.1)
	Okole	45 (28.5)
	Plastic container	62 (39.2)
	Welki	36 (22.8)
	Wooden bucket	1 (0.6)
Hand washing	No	141 (89.2)
	Yes	17 (10.8)
Milking utensil cleaning	No	137 (86.7)
	Yes	21 (13.3)
Udder preparation	No	152 (96.2)
	Yes	6 (3.8)
Udder hygiene	Relatively clean	133 (84.2)
	Visibly dirty	25 (15.8)
Restraining method	Rope tying the hock	17 (10.8)
	Manually handled	141 (89.2)
Calf suckles	Yes	79 (50.0)
	No	79 (50.0)
Milker	Boy	15 (9.5)
	Girl	2 (1.3)
	Man	8 (5.1)
	Woman	27 (17.1)
	Boy and woman	11 (7.0)
	Boy and girl	22 (13.9)
	Boy and man	13 (8.2)
	Boy and two girls	1 (0.6)
	Boy, man and woman	1 (0.6)
	Boy, girl and man	2 (1.3)
	Boy, girl and woman	18 (11.4)
	Girl and woman	4 (2.5)
	Man and girl	1 (0.6)
	Man and woman	11 (7.0)
	Two boys and a man	2 (1.3)
	Two boys	14 (8.9)
	Two boys and a woman	2 (1.3)
	Two men	1 (0.6)
	Two women	2 (1.3)
	Two girls	1 (0.6)
Fecal consistency	Fluid	1 (0.6)
	Hard	76 (48.1)
	Normal	77 (48.7)
	Soft	4 (2.5)
Teat lesion	Yes	3 (1.9)
	No	155 (98.1)

**Table 3:** Effects of various risk factors on the prevalence of *E. coli* O157:H7 and *Salmonella* in the feces of dairy cows in the Borana pastoralist community.

Parameter	Categories	No. Sampled	*E. coli* O157:H7		*Salmonella*	
			No. Positive	*p*-Value *	No. Positive	*p*-Value *
District	Dubuluk	50	0	0.02	3	0.019
	Yabello	66	6		10	
Village	Colqasa	3	1	0.426	0	0.230
	Dharito	31	2		4	
	Dida Yabello	31	3		6	
	Igo	10	0		1	
	Malicha Huka	10	0		1	
	Surupha	3	0		1	
Animal species raised	Cattle, camel	3	0	1.00	1	0.2
	Cattle, camel, goat, sheep	103	5		8	
	Cattle, goat, sheep	28	1		3	
	Cattle, sheep	2	0		1	
Age in years	5	17	1	0.575	1	0.665
	6	42	3		3	
	7	33	0		2	
	8	41	1		6	
	9	4	0		1	
	10	10	1		0	
Body condition score	Good	89	5	0.641	7	0.536
	Medium	53	1		6	
Stage of lactation in months	1	3	0	0.643	1	0.545
	2	5	1		1	
	6	7	0		1	
	12	57	5		7	
	18	20	0		1	
	24	13	0		2	
Parity number	1	38	2	0.407	4	0.645
	2	40	2		2	
	3	43	2		3	
	4	22	0		4	
	6	4	1		0	
Milking utensils	Jerrycan	14	0	0.414	1	0.939
	Okole	87	2		7	
	Plastic container	41	4		5	
Hand washing	No	123	6	0.601	12	0.468
	Yes	31	0		1	
Milking utensil cleaning	No	119	6	0.338	12	0.3
	Yes	35	0		1	
Udder preparation	No	143	6	1.00	13	0.6
Udder hygiene	Relatively clean	140	6	1.00	13	0.6
Restraining method	Rope tying the hock	105	4	1.00	9	1.00
	Manually handled	49	2		4	
Calf suckles	Yes	90	3	0.693	4	0.042
	No	64	3		9	
Milker	Woman	130	6	1.00	13	0.452
Fecal consistency	Fluid	49	1	0.398	3	0.077
	Hard	3	0		1	
	Normal	75	5		9	
Teat lesion	Yes	5	1	0.182	0	1.00
	No	149	5		13	

* Fisher’s exact test or chi-squared test was used. Categories with negative observations in both cows and camels for each bacterial species have been removed to reduce the size of the table. Readers can refer to Table 1 for detailed descriptions of the factors analyzed.

**Table 4:** Effects of various risk factors on the detection of *E. coli* O157:H7 and *Salmonella* in composite milk samples from dairy cows in the Borana pastoralist community.

Parameter	Category	No. Sampled (*n* = 154)	*E. coli* O157:H7		*Salmonella*	
			No. Positive	*p*-Value *	No. Positive	*p*-Value *
District	Dubuluk	50	0	0.089	3	0.382
	Yabello	66	4		3	
Village	Colqasa	3	1	0.154	2	<0.001
	Dharito	31	3		1	
	Igo	10	0		1	
	Surupha	3	0		2	
Animal species raised	Cattle, camel, goat, sheep	103	4	1.00	6	0.659
Age in years	6	42	1	0.623	1	0.071
	8	41	3		2	
	10	10	0		2	
	13	3	0		1	
Body condition score	Good	89	4	0.383	3	0.108
	Medium	53	0		1	
	Poor	12	0		2	
Stage of lactation in months	12	57	4	0.904	4	0.976
	18	20	0		1	
	24	13	0		1	
Parity number	1	38	1	0.415	1	0.002
	3	43	1		2	
	4	22	2		0	
	6	4	0		2	
	7	2	0		1	
Milking utensils	Okole	87	2	0.807	1	0.058
	Plastic container	41	2		4	
	Welki	3	0		1	
Hand washing	No	123	4	0.584	6	0.601
Milking utensil cleaning	No	119	4	0.575	6	0.338
Udder preparation	No	143	4	1.00	6	1.00
Udder hygiene	Relatively clean	140	4	1.00	6	1.00
Restraining method	Rope tying the hock	105	3	1.00	4	1.00
	Manually handled	49	1		2	
Calf suckles	Yes	90	3	0.642	1	0.082
	No	64	1		5	
Milker	Woman	130	4	1.00	6	1.00
Fecal consistency	Fluid	49	3	0.359	1	0.398
	Normal	75	1		5	
Teat lesion	Yes	5	1	0.125	0	1.00
	No	149	3		6	

* Fisher’s exact test or chi-squared test was used. Categories with negative observations in both cows and camels for each bacteria species have been removed to reduce the size of the table. Readers can refer to Table 1 for detailed descriptions of the factors analyzed.

**Table 5:** Effects of various risk factors on the detection of *Staphylococcus aureus* in composite milk samples from dairy cows and camels in the Borana pastoralist community.

Parameter	Categories	Cows			Camels		
		No. Tested (*n* = 119)	No. Positive	*p*-Value *	No. Tested (*n* = 130)	No. Positive	*p*-Value *
District	Dubuluk	45	14	0.110	42	19	0.036
	Elweya	8	5		13	2	
	Surupha	0	0		9	7	
	Yabello	66	17		66	30	
Village	Areri	6	3	0.051	9	2	0.256
	Buya	3	1		9	7	
	Colqasa	3	0		13	6	
	Dharito	31	4		32	14	
	Dida Yabello	31	12		12	5	
	Elweya	2	2		0	0	
	Igo	10	5		0	0	
	Jigesa	2	0		30	15	
	Jijidu	1	1		9	5	
	Lafto	21	5		12	4	
	Malicha Huka	6	2		0	0	
	Surupha	3	1		0	0	
Animal species raised	Cattle, camel, goat	6	1	0.958	4	1	0.541
	Cattle, camel, goat, sheep	82	26		122	57	
	Cattle, goat, sheep	27	9		0	0	
Age in years	5	13	3	0.782	2	2	0.285
	6	30	12		20	6	
	7	24	6		21	11	
	8	35	10		23	14	
	9	3	2		11	3	
	10	8	2		27	11	
	11	0	0		8	3	
	12	3	1		14	6	
	13	2	0		1	1	
	14	0	0		1	1	
Body condition score	Good	72	21	0.775	61	29	0.835
	Medium	38	13		50	21	
	Poor	9	2		19	8	
Stage of lactation in months	1	3	1	0.839	12	2	0.294
	2	5	2		5	4	
	3	4	1		3	1	
	4	1	1		7	3	
	5	4	1		8	3	
	6	5	2		5	3	
	7	3	1		4	0	
	8	13	2		15	8	
	9	6	2		9	3	
	10	3	1		4	1	
	11	1	1		1	0	
	12	47	12		35	19	
	18	11	4		6	2	
	24	13	5		16	9	
Parity number	1	29	8	0.415	25	9	0.594
	2	27	9		27	13	
	3	36	10		28	14	
	4	19	8		27	11	
	5	3	0		14	7	
	6	3	1		4	3	
	7	2	0		1	1	
Milking utensils	Jerrycan	10	3	0.891	1	1	0.213
	Metal cup	2	0		8	6	
	Okole	70	22		41	19	
	Plastic container	32	11		52	19	
	Welki	3	0		27	13	
Hand washing	No	101	29	0.411	119	56	0.110
	Yes	18	7		11	2	
Milking utensil cleaning	No	101	31	1.00	119	56	0.110
	Yes	18	5		11	2	
Udder preparation	No	113	35	0.666	126	57	0.628
	Yes	6	1		4	1	
Udder hygiene	Relatively clean	113	35	0.666	113	50	1.00
	Visibly dirty	6	1		17	8	
Restraining method	Rope tying the hock	76	26	0.299	9	6	0.187
	Manually handled	43	10		121	52	
Calf suckles	Yes	70	22	0.840	58	27	0.725
	No	49	14		72	31	
Milker	Boy	1	1	0.262	13	7	0.620
	Boy and woman				11	4	
	Boy and girl				16	8	
	Boy and man				13	7	
	Boy, man and woman				1	1	
	Boy, girl and woman				18	7	
	Girl	11	5		0	0	
	Girl and woman				4	3	
	Man	1	0		7	4	
	Man and woman				11	2	
	Two boys and man				2	1	
	Two boys				14	5	
	Two boys and woman				2	1	
	Two men				1	1	
	Two women				2	2	
	Woman	106	30		11	5	
Fecal consistency	Fluid	35	3	0.002	1	1	0.356
	Hard	1	0		63	26	
	Normal	63	27		64	31	
	Soft	20	6		2	0	
Teat lesion	Yes	4	1	1.00	1	1	0.446
	No	115	35		129	57	

* Fisher’s exact test or chi-squared test was used. Categories with negative observations in both cows and camels for each bacterial species have been removed to reduce the size of the table. Readers can refer to Table 2 for detailed descriptions of the factors analyzed. The blank spaces did not apply to the other animal species.

**Table 6:** Effects of the various risk factors on the detection of *Salmonella* in fecal and composite milk samples from camels in the Borana pastoralist community.

Parameter	Categories *	No. Sampled (*n* = 158)	Feces		Composite Milk	
			No. Positive	*p*-Value	No. Positive	*p*-Value
District	Dubuluk	42	2	0.516	1	1.00
	Yabello	66	2		1	
Village	Jigesa	30	2	0.756	1	1.00
	Dharito	32	1		1	
	Jijidu	9	1		0	
Animal species raised	Cattle, camel, goat	6	1	0.307	0	1.00
	Cattle, camel, goat, sheep	136	3		2	
Age in years	6	23	2	0.4	2	0.237
	8	28	2		0	
Body condition score	Good	73	3	0.234	0	0.406
	Medium	65	0		2	
	Poor	20	1		0	
Stage of lactation in months	2	5	1	0.073	0	0.924
	11	1	1		0	
	12	44	1		1	
	24	19	1		1	
Parity number	1	28	2	0.605	2	0.163
	2	35	1		0	0
	4	33	1		0	0
Milking utensils	Okole	45	1	0.714	0	0.775
	Plastic container	62	1		1	
	Welki	36	2		1	
Hand washing	No	141	4	1.00	2	1.00
Milking utensil cleaning	No	137	4	1.00	2	1.00
Udder preparation	No	152	4	1.00	2	1.00
Udder hygiene	Relatively clean	133	3	0.502	2	1.00
	Visibly dirty	25	1		0	
Restraining method	Rope tying the hock	17	1	0.369	0	1.00
	Manually handled	141	3		2	
Calf suckles	Yes	79	1	0.62	0	0.497
	No	79	3		2	
Milker	Man	8	1	0.131	0	0.639
	Boy	15	0		1	
	Boy and girl	22	1		0	
	Boy, girl and woman	18	1		1	
	Man and girl	1	1		0	
Fecal consistency	Hard	76	2	1.00	0	0.528
	Normal	77	2		2	
Teat lesion	No	155	4	1.00	2	1.00

* Only categories with positive observations are shown; for a full description of the factors, please refer to Table 3. Blank spaces in the table indicate no observation.

**Table 7:** Antimicrobial concentrations (µg/disk), interpretive categories and zone diameter (mm) breakpoints for *Enterobacteriaceae*.

Antimicrobial Agent (Code)	Concentration	Interpretation		
		Susceptible	Intermediate	Resistant
Ampicillin (AMP)	10	≥17	14–16	≤13
Chloramphenicol (CHL)	30	≥18	13–17	≤12
Ciprofloxacin (CIP)	5	≥21	16–20	≤15
Gentamicin (GEN)	10	≥15	13–14	≤12
Nalidixic acid (NAL)	30	≥18	14–18	≤13
Streptomycin (STR)	10	≥15	12–14	≤11
Tetracycline (TET)	30	≥15	12–14	≤11
Kanamycin (KAN)	30	≥18	14–17	≤13
Trimethoprim (TMP)	5	≥16	11–15	≤10

**Table 8:** Antimicrobial susceptibility test results for *E. coli* O157:H7 and *Salmonella* isolates from milk and fecal samples collected from lactating cows and camels under pastoral production system.

Antimicrobial Class	Antimicrobial Agent	*E. coli* O157:H7 (*n* = 11)			*Salmonella* (*n* = 25)		
		S (%)	I (%)	R (%)	S (%)	I (%)	R (%)
Aminoglycoside	Streptomycin	18.2	9.1	73	52	20	28
Fluoroquinolone	Nalidixic acid	100	0	0	100	0	0
Aminoglycoside	Kanamycin	64	36.4	0	88	8	4
Aminoglycoside	Gentamicin	100	0	0	100	0	0
Fluoroquinolone	Ciprofloxacin	100	0	0	100	0	0
Phenicols	Chloramphenicol	100	0	0	96	4	0
Beta-Lactam	Ampicillin	0	0	100	0	0	100
Tetracycline	Tetracycline	27.3	9.1	64	88	0	12
Folate pathway inhibitor	Trimethoprim	82	0	18.2	100	0	0

S = Susceptible; I = Intermediate; R = Resistant.

**Table 9:** Antimicrobial resistance profiles of *E. coli* O157:H7 and *Salmonella* isolates from cow and camel fecal and milk samples from the Borana pastoral community.

No. of Drug Classes	Resistance Profile (No. of Isolates)			
	*E. coli* O157:H7		*Salmonella*	
	Cows (*n* = 10)	Camels (*n* = 1)	Cows (*n* = 19)	Camels (*n* = 6)
One		AMP (1)	AMP (15)	AMP (1)
Two	AMP–STR (2)		AMP–STR (2)	AMP–STR (3)
	AMP–TET (1)		AMP–TET (1)	
			AMP–KAN (1)	
Three	AMP–STR–TET (5)			AMP–STR–TET (2)
	AMP–STR–TMP (1)			
	AMP–TET–TMP (1)			

AMP: Ampicillin; TET: Tetracycline; STR: Streptomycin; TMP: Trimethoprim; KAN: kanamycin.

## Data Availability

The data can be obtained upon request from the corresponding author.

## Supplementary Materials

The following supporting information can be downloaded at: https://www.mdpi.com/article/10.3390/antibiotics13010026/s1, Table S1: Inhibition zone diameter of antimicrobial disks used to determine the antimicrobial susceptibility of E. coli and Salmonella isolates from dairy cows and camels in the Borana pastoral community in South Oromia, Ethiopia.
